# Acid Sphingomyelinase Contributes to the Control of Mycobacterial Infection via a Signaling Cascade Leading from Reactive Oxygen Species to Cathepsin D

**DOI:** 10.3390/cells9112406

**Published:** 2020-11-03

**Authors:** Yuqing Wu, Cao Li, Huiming Peng, Ashraf Swaidan, Andrea Riehle, Barbara Pollmeier, Yang Zhang, Erich Gulbins, Heike Grassmé

**Affiliations:** 1 Department of Molecular Biology, University of Duisburg-Essen, Hufelandstrasse 55, 45122 Essen, Germany; yuqing.wu@uni-due.de (Y.W.); livenlife@163.com (C.L.); hmpeng2003@hust.edu.cn (H.P.); ashrafswaidan89@gmail.com (A.S.); andrea.riehle@uni-due.de (A.R.); barbara.pollmeier@uni-due.de (B.P.); erich.gulbins@uni-due.de (E.G.); 2 Department of Pharmacy, Beijing Tiantan Hospital, Capital Medical University, 119 4th South Ring, Fengtai District, Beijing 100050, China; 3 Department of Anatomy, School of Basic Medicine, Tongji Medical College, Huazhong University of Science and Technology, Wuhan 430074, China; 4 Department of Pharmacological and Pharmaceutical Sciences, University of Houston, 3517 Cullen Blvd, Houston, TX 77204-5056, USA; yzhan219@central.uh.edu; 5 Department of Surgery, University of Cincinnati, 231 Albert Sabin Way, Cincinnati, OH 45267, USA

**Keywords:** *Mycobacterium bovis* Bacillus Calmette-Guérin (BCG), acid sphingomyelinase (Asm), ceramide, macrophages, cathepsin D (CTSD), reactive oxygen species (ROS), granuloma

## Abstract

Tuberculosis, caused by *Mycobacterium tuberculosis*, is one of the most severe diseases worldwide. The initial pulmonary localization of the pathogen often develops into systemic infection with high lethality. The present work investigated the role of sphingolipids, specifically the function of acid sphingomyelinase (Asm) and ceramide, in infection of murine macrophages in vitro and mice in vivo with *Mycobacterium bovis* Bacillus Calmette-Guérin (BCG). In vitro, we investigated macrophages from wild-type (wt) and Asm deficient (Asm^−/−^) mice to define signaling events induced by BCG infection and mediated by Asm. We demonstrate that infection of wt macrophages results in activation of Asm, which increases reactive oxygen species (ROS) via stimulation of nicotinamide adenine dinucleotide phosphate (NADPH) oxidase. ROS promote BCG degradation by cathepsin D. Asm deficiency in macrophages abrogates these effects. In vivo studies reveal that wt mice rapidly control BCG infection, while Asm^−/−^ mice fail to control the infection and kill the bacteria. Transplantation of wt macrophages into Asm^−/−^ mice reversed their susceptibility to BCG, demonstrating the importance of Asm in macrophages for defense against BCG. These findings indicate that Asm is important for the control of BCG infection.

## 1. Introduction

Sphingomyelinases are enzymes classified according to the optimal pH for their activity: acid, neutral, or alkaline [1]. Sphingomyelinases catalyze the hydrolysis of sphingomyelin to generate ceramide [2], which is a central molecule in modulating membrane biophysical properties and is involved in various cellular process, such as apoptosis and inflammation, as well as several pathologies and diseases [3]. Acid sphingomyelinase generates ceramide in the outer leaflet of the plasma membrane and in lysosomes. Ceramide molecules reorganize the cell membrane, resulting in the formation of large, distinct ceramide-enriched membrane domains that serve to cluster and aggregate activated receptor molecules. Receptor clustering allows the amplification of receptor signaling and thereby efficient signal transduction into the cells [4]. Acid sphingomyelinase and ceramide have been shown to be crucially involved in the host response to various bacteria, including pathogenic mycobacteria, several viruses, and some parasites [5,6,7,8,9,10,11,12,13,14]. Deficiency of acid sphingomyelinase often leads to increased susceptibility of the host to pathogens, such as *Pseudomonas aeruginosa*, *Listeria monocytogenes*, *Salmonella typhimurium*, and *Staphylococcus aureus* via distinct mechanisms including failure of internalization, loss of fusion of bacteria-containing phagosomes with lysosomes, or a defect in the nicotinamide adenine dinucleotide phosphate (NADPH)-mediated release of reactive oxygen species (ROS) [7,8,9,11,15]. In order to effectively kill phagocytosed bacteria within phagolysosomes, various degradative enzymes are required, such as cathepsins, proteases, lysozymes, and lipases. Previous studies provide evidence for a specific interaction of acid-sphingomyelinase-derived ceramide with cathepsin D (CTSD), leading to enhanced enzymatic activity and proteolytic activation of proteins to be secreted [16].

Diseases caused by infections with pathogenic mycobacteria such as *Mycobacterium tuberculosis* and *Mycobacterium avium* are among the most common severe infections worldwide. In all countries, *Mycobacterium tuberculosis* is the primary cause of tuberculosis (TB), with more than 8 million new cases and approximately 1.6 million casualties annually [17]. Most people infected with *Mycobacterium tuberculosis* are clinically asymptomatic, a state that is referred as latent TB; about 5–10% of them can develop a severe systemic infection with high lethality [17].

The first indications that sphingomyelinases and ceramide may be involved in mycobacterial infections were reported in 2003. Anes et al. found that ceramide, sphingomyelin, sphingosine, and sphingosine 1-phosphate were involved in actin nucleation on phagosomes, thereby triggering the fusion of phagosomes with lysosomes to release antimicrobial factors, which killed mycobacteria [18]. Acid sphingomyelinase also plays a central role in phagolysosomal fusion upon infection of macrophages with *Mycobacterium avium* by modifying the steric conformation of cellular membranes [11]. A common survival strategy used by mycobacteria is to interfere with phagosome maturation and block phagosomal acidification. A recent study indicated that acid-sphingomyelinase-mediated maturation of phagosomes is regulated by neurotensin receptor 3, sortilin, and is important for controlling mycobacterial infection [19,20]. Studies using a zebrafish model described that *Mycobacterium marinum* infection in the host resulted in rapid production of mitochondrial reactive oxygen species (ROS) in infected macrophages, which was controlled by a combined interaction between mitochondrial cyclophilin D and acid sphingomyelinase [21]. These findings indicate that acid-sphingomyelinase-mediated maturation of phagosomes is important for controlling mycobacterial infection [11,19,20]. Therefore, we characterized the role of acid sphingomyelinase in *Mycobacterium bovis* Bacillus Calmette-Guérin (BCG) killing and infection in vivo and in vitro. Based on our results, we suggest a model in which acid sphingomyelinase controls mycobacterial infection by activating nicotinamide adenine dinucleotide phosphate (NADPH) oxidase and releasing reactive oxygen species (ROS). ROS promote BCG degradation via the bactericidal enzyme cathepsin D. Acid-sphingomyelinase-deficient mice failed to activate this cascade, leading to a high susceptibility to BCG infection.

## 2. Materials and Methods

### 2.1. Mice, Cells, Inhibitors

Acid-sphingomyelinase-deficient (Asm^−/−^*,* sphingomyelin phosphodiesterase 1 knockout; Smpd1^−/−^) mice and syngenic wild-type (wt) littermates were maintained on a C57BL/6J background [22]. We used Asm^−/−^ mice and their wt littermates aged only 6 to 8 weeks in order to avoid an accumulation of sphingomyelin [23]. The respective genotypes were verified by polymerase chain reaction. All mice were kept under pathogen-free conditions in the animal facility of the University of Duisburg-Essen in accordance with the criteria of the Association of Laboratory Animal Sciences. In vivo infections were approved by the Landesamt für Natur, Umwelt und Verbraucherschutz (LANUV); animal grants G 903/0, No. 887-501034.09, and G 1691/18, No. 81-02.04.2018.A192.

The in vitro experiments were carried out with freshly isolated bone-marrow-derived macrophages (BMDMs) from wt or Asm^−/−^ mice. To obtain BMDMs, mice were sacrificed, and femurs and tibias were rinsed with minimum essential medium (MEM; Thermo Fisher Scientific, Waltham, MA, USA) enriched with 10% fetal bovine serum (Thermo Fisher Scientific, Waltham, MA, USA), 10 mM HEPES (pH 7.4; Roth GmbH, Karlsruhe, Germany), 2 mM L-glutamine, 1 mM sodium pyruvate, 100 μM nonessential amino acids, 100 U/mL penicillin, and 100 μg/mL streptomycin (Thermo Fisher Scientific, Waltham, MA, USA). Isolated cells were passed through a 23-G needle to obtain single cells, which were cultured for 24 h in small tissue-culture flasks. Culturing of BMDMs has been previously described in detail [15]. Briefly, cells were washed, and 3 × 10^4^ or 1.2 × 10^5^ non-adherent cells were cultured in 24- or 6-well plates in MEM with 20% L-cell supernatant as a source of macrophage colony-stimulating factor (M-CSF). Fresh MEM/L-cell supernatant medium was applied after 4 days of culture. Macrophages matured within the next 6 days and were used on day 10 of culture.

In the present study, we used the cathepsin D inhibitor Pepstatin A (Sigma-Aldrich, Steinheim, Germany) or the ROS inhibitor Apocynin (Abcam, Cambridge, UK), both at a concentration of 10 mM. Cells were pre-incubated with inhibitor for 1 h and then infected as described.

### 2.2. Infection Experiments

The in vivo infections of Asm^−/−^ and wt mice and the in vitro infections of bone-marrow-derived macrophages (BMDMs) were performed with green fluorescent protein-expressing BCG (GFP-BCG) [24,25]. To construct the GFP-BCG strain, BCG were transformed with the dual reporter plasmid pSMT3L × EGFP [25]. For infection experiments, bacteria were shaken at 120 rpm at 37 °C in Erlenmeyer flasks with 10 mL Middlebrook 7H9 Broth supplemented with glycerol (BD Biosciences, Heidelberg, Germany) and 50 µg/mL Hygromycin B to maintain GFP plasmids. After 5 to 7 days of culture, the bacteria were used for infection experiments. To this end, the suspended bacteria were collected by centrifugation at 880× *g* for 10 min. In order to separate clumped BCG, the bacterial pellet was resuspended in HEPES/saline buffer (H/S) consisting of 132 mM NaCl, 1 mM CaCl_2_, 0.7 mM MgCl_2_, 20 mM HEPES (pH 7.3), 5 mM KCl, and 0.8 mM MgSO_4_ and vortexed for 5 min at high speed. Samples were bath-sonicated for 5 min at 4 °C and passed 10 times through a syringe with an 0.8 mm diameter needle. Unseparated clumps of bacteria were removed by centrifugation at 220× *g* for 2 min. The supernatant containing single GFP-BCG was carefully collected. The bacterial number was calculated with a 100× oil lens of an inverted fluorescence microscope (DMIRE2; Leica Microsystems, Wetzlar, Germany).

For in vitro assays, BMDMs were kept in MEM/10 mM HEPES (pH 7.4) and either left uninfected or infected with GFP-BCG for the indicated time at a ratio of bacteria-to-host cells (multiplicity of infection, MOI) of 5:1 to 10:1. In order to achieve increased interaction between bacteria and host cells and to obtain synchronous infection conditions, the bacteria were centrifuged onto the cells for 8 min at 55× *g*. The end of centrifugation was defined as the starting point of infection. The infection was terminated either by fixation or lysis, as described below. For in vivo infections, bacteria were prepared as described above and then pelleted at 2.240× *g* for 10 min. BCG were resuspended in 0.9% NaCl and 1 × 10^7^ colony-forming units (CFU) of bacteria in 100 μL were injected intravenously into mice.

### 2.3. Discrimination between Intra- and Extracellular GFP-BCG

To discriminate between binding and internalization of GFP-BCG, we carried out reduction in green fluorescence of Trypan blue quenched bacteria by excitation energy transfer [26,27]. To this end, we washed infected or uninfected bone-marrow-derived macrophages in cold phosphate buffered saline (PBS) buffer (137 mM NaCl, 2.7 mM KCl, 10 mM Na_2_HPO_4_, 2.0 mM KH_2_PO_4_; pH adjusted to 7.4) and incubated them in PBS on ice for 15 min. Cells were harvested, normalized to a concentration of 1 × 10^6^ cells/50 µL in PBS, and either fixed in 4% paraformaldehyde (PFA; Sigma-Aldrich, Steinheim, Germany) for 10 min at room temperature or processed to quench adherent bacteria. To quench the fluorescence of adherent GFP-BCG, 500 µL Trypan blue (0.4% *w*/*v* in PBS; Corning Inc., Corning, NY, USA) was added for 1 min, followed by washing twice in PBS and fixation in 4% PFA for 10 min at room temperature. Quenching with Trypan blue reduced the GFP fluorescence of only adherent bacteria (not internalized bacteria) by excitation energy transfer [26,27]. Bacterial binding (without Trypan blue treatment) and internalization (with Trypan blue treatment) were analyzed by Attune NxT flow cytometry (Thermo Fisher Scientific, Waltham, MA, USA) and FlowJo software v10 (FlowJo LLC, Ashland, OR, USA).

### 2.4. Depletion and Transplantation of Bone-Marrow-Derived Macrophages

Liposomes were purchased from Liposoma BV, Netherlands, stored at 4 °C, and calibrated to room temperature for 2 h before injection. For bone-marrow-derived macrophage (BMDM) depletion, clodronate, or PBS liposomes (200 µL) were infused intravenously. For reconstitution, macrophages were generated by culturing BMDMs as previously described. Two days after depletion, mice were left untreated or intravenously injected with 10^6^, 5 × 10^6^, or 10^7^ macrophages. Mice were sacrificed 3 days after transplantation of macrophages. Liver, spleen, and bone marrow were collected for detection of macrophages by Attune NxT flow cytometer (Thermo Fisher Scientific, Waltham, MA, USA). Briefly, cell suspension was harvested from bone marrow, liver, and spleen and adjusted to a concentration of 1 × 10^6^ cells/50 µL in PBS. Cells were incubated for 30 min at 4 °C with anti-mouse CD16/32 antibody (Biolegend, San Diego, CA, USA) to block Fcγ receptors, followed by incubation with primary fluorochrome-conjugated antibodies specific to mouse PE-Gr-1 (Ly6G/C; clone RB6-8C5), Brilliant Violet-CD115 (clone AFS98), Pacific Blue-CD11b (clone M1/70), PerCP-CD3 (clone 145-2C11), PerCP-CD45R/B220 (clone RA3-6B2), PerCP-Ter119 (clone TER-119), APC-F4/80 (clone BM8), or PE/Cy7-NK1.1 (clone PK136), all from Biolegend, San Diego, CA, USA for 45 min at 4 °C. After washing 2 times with PBS, cells were resuspended in PBS and analyzed with an NxT flow cytometer (Thermo Fisher Scientific, Waltham, MA, USA). Stained cell suspensions were analyzed with multiparameter FlowJo software v10 (FlowJo LLC, Ashland, OR, USA).

### 2.5. Transfection of Bone-Marrow-Derived Macrophages

The all-in-one mouse CTSD gRNA/CRISPR-cas9 plasmid and CRISPR-cas9 control plasmid (without CTSD-gRNA) were purchased from GenScript (Piscataway, NJ, USA). Plasmids were transformed to DH5α-competent cells and amplified in LB medium (Roth GmbH, Karlsruhe, Germany) containing ampicillin (Sigma-Aldrich, Steinheim, Germany) overnight at 37 °C with shaking at 225 rpm. The bacteria were harvested, and plasmids were extracted and purified using a Qiagen plasmid extraction and Midi purification kit (Qiagen, Hilden, Germany) following the manufacturer’s instructions. The purified CTSD gRNA/CRISPR-Cas9 plasmid and CRISPR-Cas9 control plasmid (without CTSD gRNA) were transfected with Lipofectamine 3000 (Invitrogen, Carlsbad, CA, USA) into mature bone-marrow-derived macrophages according to the procedure provided by the company. Transfected cells with segments containing either control or CTSD gRNA were selected by adding puromycin (6 μg/mL; Thermo Fisher Scientific, Waltham, MA, USA) and were used 2–3 days after transfection for infection in puromycin-free medium.

### 2.6. Determination of Acid Sphingomyelinase Activity

For determination of Asm activity in macrophages, we performed a recently described assay that makes use of green fluorescent BODIPY FL C_12_-sphingomyelin (Thermo Fisher Scientific, Waltham, MA, USA) as a substrate for the acid sphingomyelinase [28]. For this purpose, cells were left uninfected or infected for the indicated time, harvested, and lysed in 250 mM sodium acetate (Sigma-Aldrich, Steinheim, Germany) with 1% Nonidet P-40 (pH 5.0; Sigma-Aldrich, Steinheim, Germany) for 5 min on ice. Cells were further disrupted by sonification for 10 min in an ice bath sonicator (Bandelin Electronic, Berlin, Germany). Aliquots were taken for protein measurement by a Bradford protein assay (BioRad, Feldkirchen, Germany), and 5 µg of protein in 20 μL lysis buffer was added to 250 mM sodium acetate (pH 5.0) containing 100 pmol green fluorescent BODIPY-FL_C12_-sphingomyelin. The samples were shaken for 1 h at 300 U and 37 °C, and the reaction was stopped by adding 1 mL of chloroform/methanol (2:1, *v*/*v*), followed by centrifugation at 14,000 rpm for 5 min. The lower phase was dried in a SpeedVac Concentrator (Thermo Fisher Scientific, Waltham, MA, USA), resuspended in chloroform/methanol (2:1, *v*/*v*), spotted on a thin-layer chromatography (TLC) plate (Merck, Darmstadt, Germany), and separated with chloroform/methanol (80:20, *v*/*v*). For analysis, the samples were scanned with a Typhoon FLA 9500 laser scanner (GE Healthcare Life Sciences, Freiburg, Germany) and quantified with ImageQuant software (GE Healthcare Life Sciences, Freiburg, Germany).

### 2.7. Western Blots

Infection of bone-marrow-derived macrophages (BMDMs) was carried out for the indicated time. Both infected and uninfected cells were washed in cold H/S buffer and lysed for 5 min on ice in 125 mM NaCl, 25 mM TrisHCl (pH 7.4), 10 mM ethylenediaminetetraacetic acid (EDTA), 10 mM sodium pyrophosphate, and 3% NP-40 supplemented with 10 µg/mL aprotinin/leupeptin (A/L). Cell lysates were pelleted by centrifugation for 10 min at 10.510× *g*. The supernatants were collected, added to 5× sodium dodecyl sulfate (SDS) sample buffer, and boiled for 5 min at 95 °C. Proteins were separated by 8.5% to 12.5% SDS polyacrylamide gel electrophoresis (SDS-PAGE) and transferred to nitrocellulose membranes (Protran Premium 0.2 µm; GE Healthcare, Life Sciences, Freiburg, Germany) for 2 h at 4 °C (80 V). The blots were washed with PBS and blocked for 1 h at room temperature in Starting Block Tris-buffered saline (TBS) buffer (Thermo Fisher Scientific, Waltham, MA, USA). After 2 additional washes in PBS, they were then incubated overnight at 4 °C in blocking buffer with specific primary antibodies against mature cathepsin D (Santa Cruz Biotechnology, Dallas, TX, USA), p47phox (mouse) (Merck, Darmstadt, Germany), or actin (Santa Cruz Biotechnology, Dallas, TX, USA) at 1:1000 dilution. After being subjected to 6 washing steps in TBS/Tween, blots were incubated for 1 h at room temperature in TBS/Tween containing 10% blocking buffer with alkaline phosphatase (AP)-conjugated secondary antibodies (Santa Cruz Biotechnology, Dallas, TX, USA). Samples were washed extensively and developed with CDP-Star substrate (Perkin Elmer, Rodgau, Germany). For in vivo measurement of cathepsin D expression, mice were sacrificed and tissues were removed, homogenized, and processed, as described above.

### 2.8. Measurement of Superoxide Production

Superoxide production was measured by electron spin resonance (ESR), as previously described [29]. In brief, 10^6^ cells were infected with GFP-BCG for the indicated time, the medium was removed, and the cells were scraped into 20 mM HEPES (pH 7.5), 1 mM EDTA, and 255 mM sucrose then shock-frozen in liquid nitrogen. Proteins were isolated and resuspended with modified Krebs-HEPES buffer containing deferoxamine (100 μM; Sigma) and diethyldithiocarbamate (5 μM; Sigma-Aldrich, Steinheim, Germany). A spin trap with 1-hydroxy-3-methoxycarbonyl-2,2,5,5-tetramethylpyrrolidine (1 mM final concentration; Noxygen Science Transfer and Diagnostics, Elzach, Germany) was then added to the mixture in the presence or absence of manganese-dependent superoxide dismutase (SOD, 200 U/mL; Sigma-Aldrich, Steinheim, Germany). The mixture was loaded into glass capillaries and immediately kinetically analyzed for O_2_^.^ formation for 10 min. The SOD-inhibited fraction of the signal was used to calibrate the system. The ESR settings were as follows: biofield, 3350; field sweep, 60 G; microwave frequency, 9.78 GHz; microwave power, 20 mW; modulation amplitude, 3 G; points of resolution, 4096; receiver gain, 100; and kinetic time, 10 min. The ESR signal strength was recorded in arbitrary units, and the final results were expressed as fold change from control strength, as previously described [30].

### 2.9. Immunocytochemistry

Bone-marrow-derived macrophages (BMDMs) were grown on coverslips and were infected or left uninfected. After fixation in 1% paraformaldehyde (PFA; Sigma-Aldrich, Steinheim, Germany) for 15 min at room temperature, they were washed in PBS (pH 7.4) for further staining. Cells were permeabilized with 0.1% Triton X-100 (Sigma-Aldrich, Steinheim, Germany) in PBS (pH 7.4) for 10 min at room temperature, washed once with H/S and once with H/S with 0.05% Tween-20 (Sigma-Aldrich, Steinheim, Germany), and blocked for 45 min in H/S supplemented with 5% fetal calf serum (FCS; Thermo Fisher Scientific, Waltham, MA, USA). BMDMs were washed 3 times in H/S with 0.05% Tween-20 and incubated for 45 min with cathepsin D (R&D, Minneapolis, MN, USA) or rabbit-anti-p47phox (mouse) antibodies (Merck, Darmstadt, Germany) in H/S supplemented with 1% FCS (Thermo Fisher Scientific, Waltham, MA, USA). Cells were washed 3 times in H/S with 0.05% Tween-20 and incubated with secondary antibodies corresponding to the primary antibodies for 45 min (final concentration of all antibodies, 1.5 μg/mL, diluted in 5% FCS/PBS; all antibodies from Jackson Immuno Research, Europe Ltd., Cambridgeshire, UK). To confirm the specificity of fluorescent staining, samples were incubated with secondary antibody controls. After 3 further washes in H/S with 0.05% Tween-20 and a final wash with H/S, cells were mounted on glass microscope slides with Mowiol (Kuraray Specialities Europe GmbH, Frankfurt, Germany). Cells were examined with a Leica TCS SP5 confocal microscope (Leica Microsystems, Wetzlar, Germany). For quantification of co-localization between GFP-BCG and CTSD or p47phox, randomly selected fields were chosen and at least 50 bacteria/sample were analyzed. To acquire the percentage of bacterial co-localization, the number of GFP-BCGs that co-localized with CTSD or p47phox was divided by total number of GFP-BCGs, and the results were multiplied by 100.

### 2.10. Histopathologic Assessment

After the indicated infection time, mice were sacrificed by cervical dislocation, and livers and spleens were removed. Tissues were embedded in Tissue-Tec (Sakura Finetek USA, Torrance, CA, USA) and shock-frozen in liquid nitrogen, and 6 µm thick sections were cut with a cryotome (CM1850 UV, Leica Microsystems, Wetzlar, Germany). For staining, sections were thawed, air-dried for 5 min, and fixed in ice-cold acetone for 10 min. Fluorescent visualization of mycobacteria was performed with the Truant TB Fluorescent Stain Kit (BD Difco, Becton Dickinson, Franklin Lakes, NJ, USA) according to the manufacturer’s instructions. Evaluation of bacterial distribution was carried out with an inverted fluorescence microscope or a confocal microscope (DMIRE2; Leica Microsystems, Wetzlar, Germany). For hematoxylin and eosin (H&E) staining, liver and spleen sections were prepared as above and stained for 20 min with Mayer’s hemalum solution (Roth GmbH, Karlsruhe, Germany). Samples were washed in water for 15 min, stained with 1% eosin solution for an additional 2 min, and washed again with water. After dehydration in ethanol, the samples were embedded in Eukitt mounting medium (Sigma-Aldrich, Steinheim, Germany) and analyzed with a Leica DMIRE2 microscope (Leica Microsystems, Wetzlar, Germany). Granuloma formation was examined with a 20× lens and an inverted fluorescence microscope.

For visualization of cathepsin D and murine macrophages in tissue, fixed sections were stained with antibodies against cathepsin D (Santa Cruz, Dallas, TX, USA) and corresponding Cy3-coupled secondary antibodies, APC-F4/80 (Biolegend, San Diego, CA, USA), as described above.

### 2.11. Quantification of Bacterial Numbers

To quantify mycobacteria in tissue, we removed livers and spleens from infected mice and added 5 mg/mL saponin (Serva Electrophoresis GmbH, Heidelberg, Germany) in H/S for the release of intracellular bacteria. Tissue was homogenized in a loose Dounce homogenizer (Braun, Kronberg, Germany) and incubated for 30 min at 37 °C in a thermomixer for the release of intracellular bacteria. Samples were centrifuged for 2 min at 220× *g*, resuspended in PBS, and plated on Middlebrook 7H10 agar plates supplemented with oleic acid (OADC; BD Biosciences, Heidelberg, Germany) to determine colony-forming units (CFUs). To determine the CFUs of BCG in macrophages, infected cells were washed once with MEM/10 mM HEPES (pH 7.4) after the indicated infection time to remove nonadherent bacteria, and then lysed in 3 mg/mL saponin for 30 min at 37 °C. We plated 100 µL aliquots and counted bacterial CFUs after the plates had been incubated for approximately 2 weeks in a humidified 37 °C atmosphere.

### 2.12. Statistical Analysis

All data were obtained from independent measurements and expressed as arithmetic means ± standard deviation (SD). Data were tested with the David–Pearson–Stephens test for normal distribution. Statistical analysis was performed with Student’s *t*-test for single comparisons and ANOVA for multiple comparisons. GraphPad Prism statistical software 6 (GraphPad Software, La Jolla, CA, USA) was used for analysis.

## 3. Results

### 3.1. Acid-Sphingomyelinase-Deficient Mice Are More Susceptible to BCG Infection than Wild-Type mice

**Figure 1:**
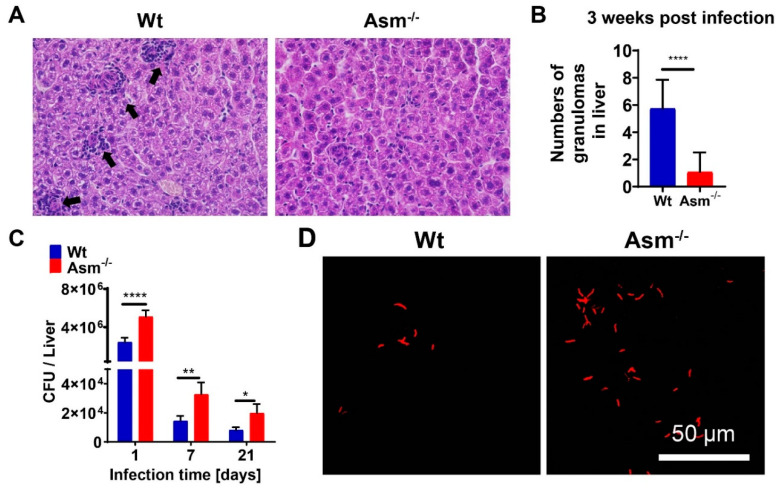
Acid-sphingomyelinase-deficient mice are more susceptible to BCG infection. Wild-type (wt) mice and acid-sphingomyelinase-deficient (Asm^−/−^) mice were intravenously infected with 10^7^ colony-forming units (CFU) of *Mycobacterium bovis* Bacillus Calmette-Guérin (BCG) for 1, 7, or 21 days. (**A**,**B**) Cryosections from liver tissues after 3 weeks of infection were stained with hematoxylin and eosin (H&E) and analyzed by light microscopy with a 20× lens. Arrows indicate granulomas. Pictures are representative of at least 3 independent experiments. The number of granulomas was determined by counting them in 10 serial sections. Shown is the mean ± SD of granuloma per section, n = 4, *t*-test. (**C**) The total number of BCG in liver tissue homogenates after 1, 7, or 21 days of infection was determined by CFU assays. (**D**) BCG inside liver tissues after 3 weeks of infection were visualized by Truant staining with a 100× lens. Scale bar = 50 µm. Shown are means ± SD of numbers of bacteria (**C**), n = 4, ANOVA, statistical significance was set as **** *p* < 0.0001, ** *p* < 0.01, * *p* < 0.05 or representative pictures of at least 3 independent experiments (**D**).

First, we examined whether acid sphingomyelinase (Asm) was important for BCG infection in vivo. To this end, we infected wild-type (wt) and acid-sphingomyelinase-deficient (Asm^−/−^) mice intravenously with BCG and determined granuloma formation and bacterial numbers in tissues 1, 7, and 21 days after infection. In wt mice, we observed multiple granulomas in livers after 3 weeks of infection (Figure 1A,B). The number of granulomas was much lower in Asm-deficient mice (Figure 1A,B). As for bacterial load, we found a significantly lower hepatic burden of BCG in wt mice compared to Asm-deficient mice (Figure 1C,D). Thus, Asm deficiency resulted in a lack of control of the bacterial infection in the liver at all observed time points (Figure 1C,D). Similar results were obtained for spleens after 1 day and 3 weeks of infection, but no significant difference after 3 days or 1 week of infection (Figure S1). These findings indicate rapid control of bacterial infection in wt mice in the early stages and less efficient bacterial killing in Asm-deficient mice.

### 3.2. Acid Sphingomyelinase Deficiency Impairs BCG Killing but Not Internalization by Macrophages

**Figure 2:**
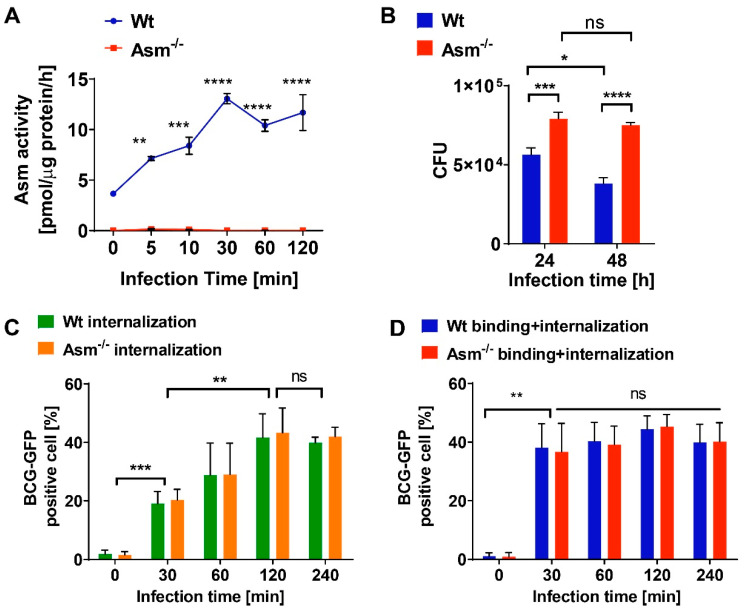
Acid sphingomyelinase deficiency impairs BCG killing but not internalization by macrophages. Bone-marrow-derived macrophages from wild-type and acid-sphingomyelinase-deficient (Asm^−/−^) mice were infected with GFP-BCG for the indicated time and (**A**) Asm activity and (**B**) colony-forming unit (CFU) assays were performed. (**C**) Bacterial internalization and (**D**) binding + internalization were investigated by flow cytometry of 10,000 cells/sample, with noninfected cells used as control. Shown are means ± SD, n = 3, ANOVA from 3 independent experiments, **** *p* < 0.0001, *** *p* < 0.001, ** *p* < 0.01, * *p* < 0.05. ns = no significant.

The rapid acid sphingomyelinase (Asm)-dependent BCG control in wild-type (wt) mice versus the inability of early BCG clearance in Asm-deficient mice in vivo suggests the involvement of phagocytes. As professional phagocytes, macrophages are the first line of defense in bacterial infection [31]. Therefore, we isolated bone-marrow-derived macrophages from wt and Asm-deficient mice and infected them with BCG to investigate the importance of Asm in BCG infection at the early stages. We observed an increase in Asm activity in wt cells upon BCG infection over time, with a maximum after 30 min of infection (Figure 2A). CFU assays revealed that wt macrophages contained 30–40% less intracellular bacteria than Asm-deficient macrophages after 24 and 48 h of infection, and bacterial number was maintained in Asm-deficient macrophages, indicating that these macrophages failed to eliminate bacteria (Figure 2B).

To investigate whether the higher bacterial load in Asm-deficient cells was caused by defective BCG internalization by macrophages, bacterial internalization was examined by analyzing the GFP signal in infected cells via FACS. To avoid signals from GFP-BCG, which only bound to cells but were not internalized, we applied Trypan blue quenching. Trypan blue can absorb the light emitted from GPF but cannot penetrate intact cells [26,27]. Therefore, cells treated and fixed by Trypan blue exhibited GFP signals only from internalized GFP-BCG, as shown in Figure 2C (internalization). In contrast, cells without Trypan blue treatment represented cells containing both adherent and internalized GFP-BCG, as shown in Figure 2D (binding + internalization). Similar results were obtained after FACS analysis with pHrodo pre-stained BCG (Figure S2A). In addition, by microscopic quantification of bacteria via GFP fluorescence, we observed that the number of GFP-expressing BCG was similar between wt and Asm-deficient macrophages within the first 3 h after infection, whereas at 6 h after infection, wt macrophages contained significantly less GFP-expressing BCG than Asm-deficient cells (Figure S2B,C).

The results revealed that Asm-expressing or -deficient macrophages equally internalized BCG, but the bacterial load in Asm-deficient macrophages was increased in comparison to wt cells (Figure 2B and Figure S2). Collectively, these data suggest that wt macrophages can eliminate BCG, while Asm-deficient macrophages fail to kill BCG after uptake.

### 3.3. Acid Sphingomyelinase Determines Expression of Cathepsin D, Which Is Essential for BCG Degradation

To understand the mechanism of acid sphingomyelinase (Asm) deficiency mediating increased infection susceptibility, we first tested whether the maturation of phagosomes upon BCG infection was defective in Asm-deficient cells. By staining the late endosomal markers Rab7 and Lamp1, we found that the maturation of phagosomes was not impaired in Asm-deficient macrophages compared with wild-type (wt) cells, as there was no difference in Rab7 and Lamp1 expression upon infection (Figure S3).

**Figure 3:**
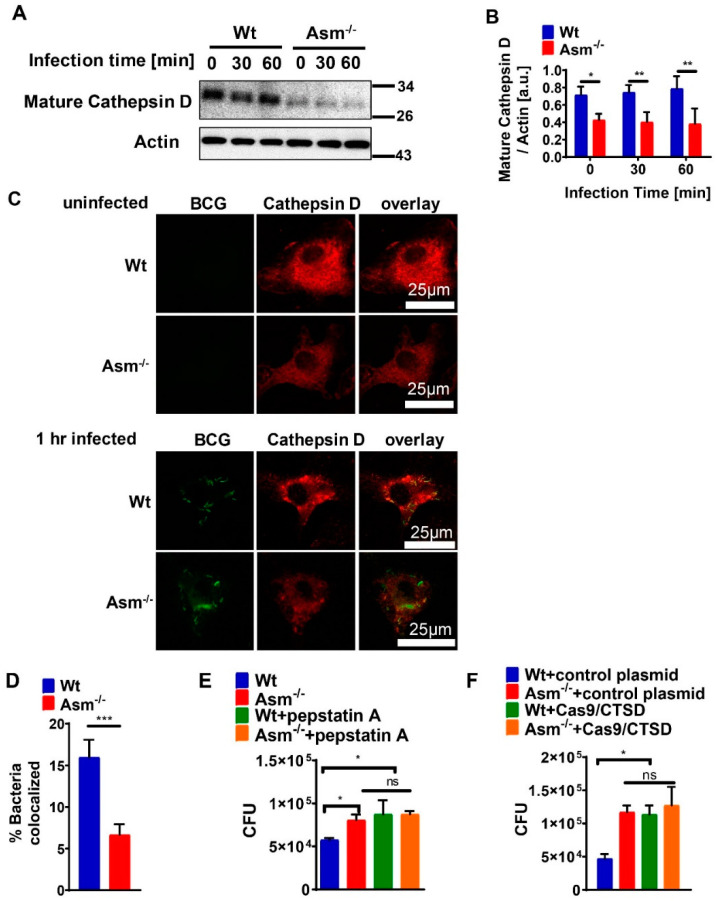
Acid sphingomyelinase determines expression of cathepsin D, which is essential for BCG degradation. (**A**) Bone-marrow-derived macrophages (BMDMs) from wild-type (wt) and Asm-deficient (Asm^−/−^) mice were infected with BCG for the indicated times or left uninfected and then subjected to Western blot analysis using antibodies against mature cathepsin D (CTSD) and β-actin. Western blot is representative of 3 independent experiments. (**B**) Expression of CTSD was normalized to actin levels and displayed as arbitrary units (a.u.) by using ImageJ. Shown are means ± SD of 3 experiments; *p*-values were calculated by ANOVA followed by Bonferroni’s multiple comparisons test, ** *p* < 0.01, * *p* < 0.05. (**C**) Wt or Asm-deficient macrophages were left uninfected or infected with GFP-BCG for 60 min, fixed, and stained with Cy3-labeled antibodies against CTSD. Samples were analyzed by confocal microscopy. Picture represents 3 independent studies. Scale bar = 25 µm. (**D**) Co-localization of GFP-BCG and CTSD was measured from at least 50 bacteria/sample and shown as mean ± SD of 3 experiments. *** *p* < 0.001 (Student’s *t*-test). (**E**) BMDMs were left untreated or pretreated with 10 μM pepstatin for 1 h and infected with BCG for 24 h. Number of BCG CFUs was determined after 2 weeks of culture. Shown are means ± SD of the CFU from 3 experiments; two-way ANOVA followed by Bonferroni’s multiple comparisons test, * *p* < 0.05. (**F**) BMDMs were transfected with either CTSD gRNA/CRISPR-cas9 plasmid or CRISPR-cas9 control plasmid, then infected with BCG for 24 h for CFU assay. Shown are means ± SD of CFU of 3 independent experiments. Quantitative analysis was performed with GraphPad and analyzed with two-way ANOVA followed by Bonferroni’s multiple comparisons test, * *p* < 0.05. ns = no significant.

We then tested the expression of different cathepsins, which have been reported to be important for Asm-dependent degradation of bacteria and serve as microbicidal factors in mycobacterial infections [16,32,33]. Our studies revealed that the expression of mature cathepsin D (CTSD) was significantly reduced in Asm-deficient macrophages in comparison to wt cells, while other cathepsins, such as B, L, K, and S, did not differ between wt and Asm-deficient macrophages (Figure 3A,B and Figure S4). Moreover, immunofluorescent staining for CTSD showed higher co-localization between CTSD and bacteria in wt macrophages after 1 h infection than in infected Asm-deficient macrophages (Figure 3C,D).

To find out whether CTSD was involved in killing BCG, we pretreated bone-marrow-derived macrophages with the CTSD inhibitor pepstatin A, infected cells, and determined the bacterial load. We observed an increase in bacterial numbers in wt macrophages to the levels observed in Asm-deficient cells, while the inhibitor did not change the bacterial burden in Asm-deficient cells (Figure 3E). These results suggest that CTSD is regulated by Asm and required for killing BCG.

To confirm the role of CTSD in killing BCG, we also transfected macrophages with an all-in-one mouse cathepsin D guide RNA CRISPR-cas9 (CTSD gRNA/CRISPR-cas9) plasmid or CRISPR-cas9 control plasmid (without CTSD-gRNA). We infected these cells for 24 h and performed CFU assays. The results showed that ablation of CTSD expression led to a higher bacterial burden in wt macrophages but had no additional effect on Asm-deficient cells (Figure 3F), supporting our notion that CTSD is essential for killing BCG in macrophages, which is absent in Asm-deficient macrophages.

### 3.4. Acid Sphingomyelinase Controls ROS Production upon BCG Infection via NADPH Oxidase

**Figure 4:**
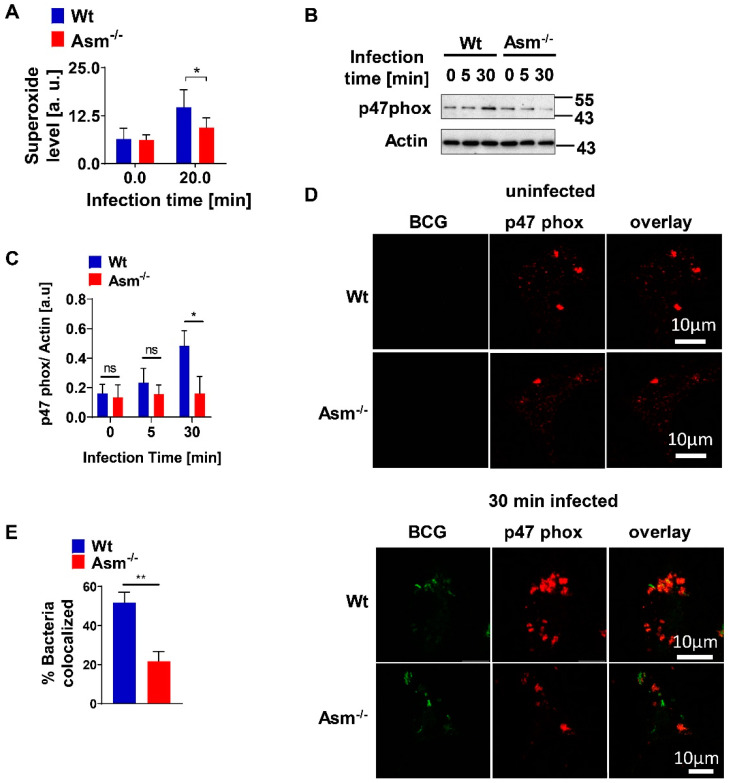
Acid sphingomyelinase controls ROS production upon BCG infection via nicotinamide adenine dinucleotide phosphate (NADPH) oxidase. Wild-type (Wt) and acid-sphingomyelinase-deficient (Asm^−/−^) bone-marrow-derived macrophages (BMDMs) were infected with BCG for the indicated time. (**A**) Superoxide production was measured by electron spin resonance. Displayed is mean ± SD of 3 experiments; ANOVA and Bonferroni’s multiple comparisons test. (**B**) Expression of NADPH oxidase subunit p47^phox^ was determined by Western blotting of lysates obtained from BCG-infected or non-infected macrophages. (**C**) Expression of p47^phox^ was normalized to actin levels and displayed as arbitrary units (a.u.) by using ImageJ. Shown are means ± SD of 3 experiments; *p*-values were calculated by ANOVA followed by Bonferroni’s multiple comparisons test. (**D**) BMDMs were infected with GFP-BCG for 30 min, fixed, and stained with Cy3-labelled p47^phox^ antibodies, and samples were analyzed by confocal microscopy. Scale bar = 10 µm. Picture represents at least 3 independent studies. (**E**) Percentage of GFP-expressing BCG that co-localized with p47 phox was measured from at least 50 bacteria/sample and presented as mean ± SD of 3 experiments. ** *p* < 0.01, * *p* < 0.05 (Student’s *t*-test). ns = no significant.

It was previously shown that acid sphingomyelinase (Asm) induces a release of reactive oxygen species (ROS), but also, in a positive loop, ROS further activate Asm [15]. We observed a rapid production of ROS in wild-type (wt) bone-marrow-derived macrophages after BCG infection compared with Asm-deficient cells (Figure 4A and Figure S5A). ROS are mainly produced via nicotinamide adenine dinucleotide phosphate (NADPH) oxidase, which is formed by five subunits. We investigated the expression of selected subunits (gp91phox, p67, p47) upon infection of macrophages with BCG and found that only p47phox showed higher expression in wt than in Asm-deficient macrophages (Figure 4B,C and Figure S5B). Accordingly, staining of NADPH oxidase subunit p47-phox showed significantly increased expression and co-localization with BCG after infection (Figure 4D,E). These effects were diminished in Asm-deficient macrophages (Figure 4D,E).

### 3.5. Inhibition of ROS Reduces Targeting of BCG to Cathepsin D

**Figure 5:**
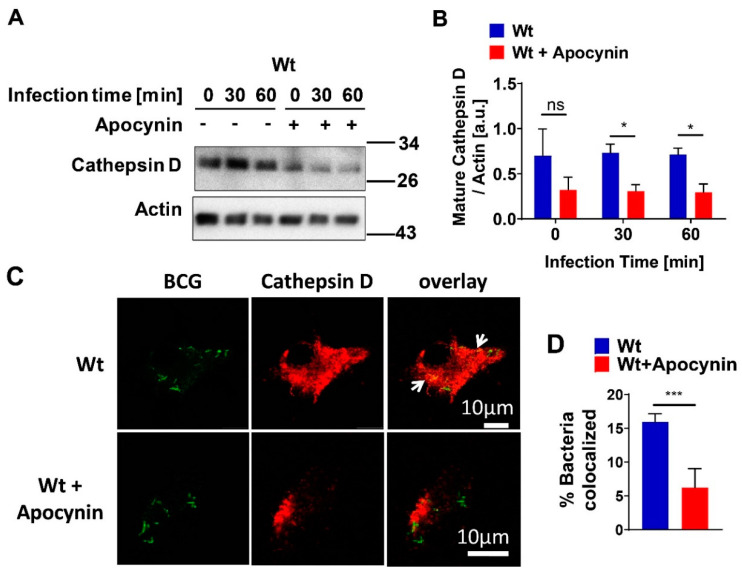
Inhibition of ROS reduces targeting of BCG to cathepsin D. (**A**) Bone-marrow-derived macrophages (BMDMs) from wild-type (wt) mice were left untreated or pretreated with 10 mΜ apocynin for 1 h, then infected with GFP-BCG for the indicated time or left uninfected and subjected to Western blotting with antibodies against mature cathepsin D (CTSD)/actin. (**B**) Expression of CTSD from wt macrophages was normalized to actin levels and quantification of CTSD arbitrary units (a.u.) was performed using ImageJ. Displayed are means ± SD of 3 experiments; *p*-values were calculated by ANOVA followed by Bonferroni’s multiple comparisons test. (**C**) Untreated or apocynin treated wt BMDMs were infected for 1 h, fixed, and stained with antibodies against mature CTSD and Cy3-labelled secondary antibodies. Scale bar = 10 µm. Arrows indicate co-localization of bacteria with CTSD. Shown are representative confocal fluorescence images from at least 3 independent experiments. (**D**) Percentage of GFP-expressing BCG and CTSD co-localization was measured from at least 50 bacteria/sample and presented as mean ± SD of 3 experiments. *** *p* < 0.001, * *p* < 0.05 (Student’s *t*-test). ns = no significant.

To investigate whether acid sphingomyelinase (Asm)-dependent ROS production was linked to cathepsin D (CTSD) maturation, we pretreated bone-marrow-derived macrophages from wild-type (wt) mice upon BCG infection with the ROS inhibitor apocynin. We found that inhibition of ROS decreased expression of mature CTSD in noninfected and infected wt cells (Figure 5A,B) and reduced co-localization of BCG with CTSD (Figure 5C,D). These results indicate that ROS promote CTSD maturation and, very likely, BCG degradation by CTSD.

### 3.6. Cathepsin D Upregulation upon BCG Infection Depends on Acid Sphingomyelinase Expression In Vivo

**Figure 6:**
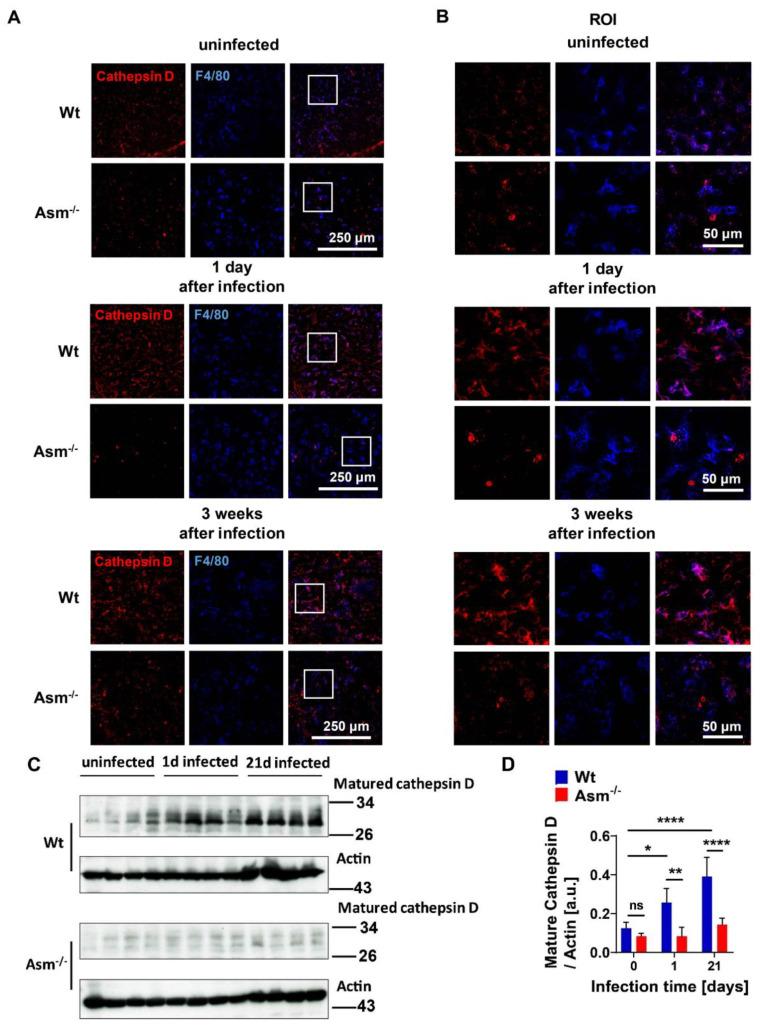
Cathepsin D upregulation upon BCG infection depends on acid sphingomyelinase expression in vivo. Wild-type (wt) and acid-sphingomyelinase-deficient (Asm^−/−^) mice were intravenously infected with 10^7^ BCG for 1 day (1d) or 3 weeks (3w) or left uninfected. (**A**–**D**) Cryosections from liver tissues were fixed and stained with antibodies against mature cathepsin D (CTSD) and corresponding Cy3-coupled secondary antibodies, APC-conjugated F4/80 antibodies, and analyzed by confocal microscopy with a 40× lens (**A**) or at higher resolution for areas of interest (ROI) (**B**). Shown are representative confocal fluorescence images from at least 3 independent experiments. Scale bar = 250 µm (**A**) or 50 µm (**B**). (**C**) CTSD expression from 4 mice/time point was visualized by Western blotting with antibodies against mature CTSD/actin. Shown are representative blots from 4 independent experiments. (**D**) CTSD expression was normalized to actin levels, and quantification of CTSD arbitrary units (a.u.) was performed by using ImageJ. Displayed are means ± SD of 4 experiments; *p*-values were calculated by ANOVA followed by Bonferroni’s multiple comparisons test, **** *p* < 0.0001, ** *p* < 0.01, * *p* < 0.05. ns = no significant.

To validate that cathepsin D (CTSD) is regulated by acid sphingomyelinase (Asm) upon mycobacterial infection in vivo, we analyzed CTSD expression in tissues from wild-type (wt) and Asm-deficient mice. Histopathologic and Western blot investigations revealed significant upregulation of mature CTSD in wt liver upon BCG infection, which was absent in the liver of Asm-deficient mice (Figure 6A–D). Additionally, co-staining of mature CTSD and F4/80 positive macrophages revealed that CTSD was expressed in hepatic macrophages (Kupffer cells), indicating that CTSD expression in wt hepatic macrophages was higher than in Asm-deficient Kupffer cells, both before and after BCG infection (Figure 6A,B). Taken together, these data confirm the results obtained in vitro with bone-marrow-derived macrophages and suggest that Asm controls CTSD, a process that leads to bacterial clearance.

### 3.7. Transplantation of Wild-Type Macrophages Reverses Susceptibility of Acid-Sphingomyelinase-Deficient Mice to BCG Infection

**Figure 7:**
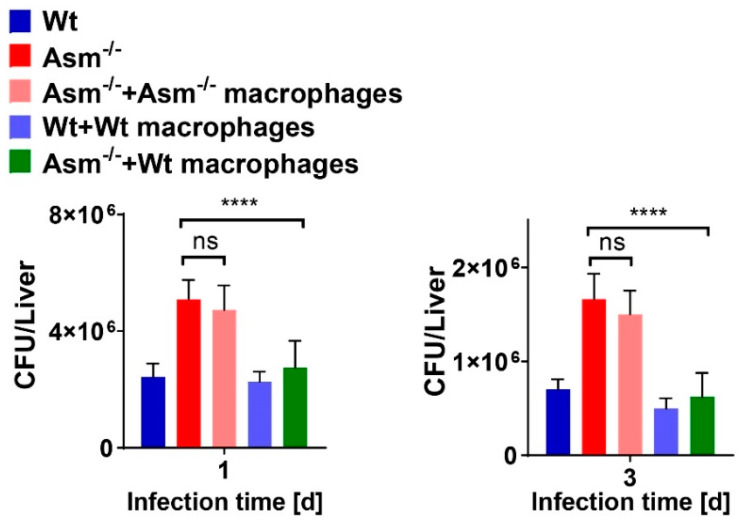
Transplantation of wild-type macrophages reverses susceptibility of acid-sphingomyelinase-deficient mice to BCG infection. Wild-type (wt) and acid-sphingomyelinase-deficient (Asm^−/−^) mice were intravenously injected with clodronate liposomes 2 days before infection and transplanted with 5 × 10^6^ wt or Asm^−/−^ bone-marrow-derived macrophages (BMDMs) via IV injection 1 day before infection with BCG. Transplanted or control mice were infected with 10^7^ BCG for 1 or 3 days. Total number of BCG in liver homogenates was determined by colony-forming unit (CFU) assays at 1 or 3 days post infection. Shown are mean ± SD; one-way ANOVA followed by a Bonferroni’s multiple comparisons test, **** *p* < 0.0001. ns = no significant.

To further prove that acid sphingomyelinase (Asm) is important for BCG infection in vivo and to clarify if macrophages are responsible for the clearance of early BCG infection, we performed in vivo transplantation experiments with clodronate liposomes and reconstitution with different macrophages. Wild-type (wt) or Asm-deficient mice were transplanted with either wt or Asm-deficient bone-marrow-derived macrophages, and tissues of those mice were subjected to CFU assays. Results reveal that transplantation of wt macrophages into Asm-deficient mice reversed their susceptibility to BCG infection in the liver (Figure 7), whereas no significant differences were obvious in the spleen (Figure S6). These results confirm the crucial role of Asm in macrophages (Figure 7).

### 3.8. Model for the Function of Asm in BCG Infection

**Figure 8:**
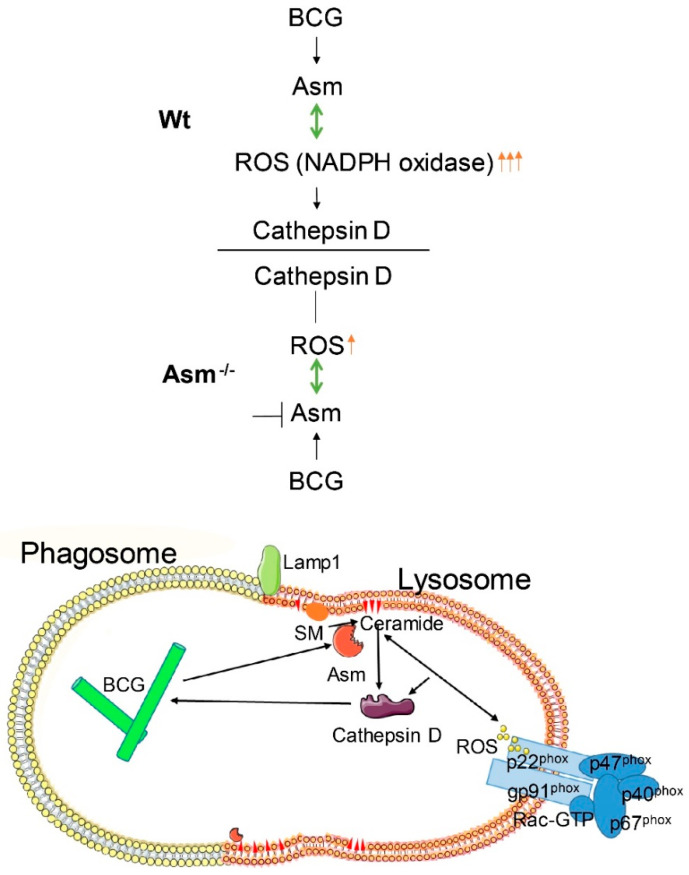
Model for Asm function in BCG infection. Infection of bone-marrow-derived macrophages (BMDMs) with *Mycobacterium bovis* Bacillus Calmette-Guérin (BCG) triggers activation of the acid sphingomyelinase (Asm)/ceramide system, which increase reactive oxygen species (ROS) via clustering of nicotinamide adenine dinucleotide phosphate (NADPH) oxidase subunit p47^phox^. ROS promote BCG degradation by the lysosomal enzyme cathepsin D (CTSD). Macrophages of Asm-deficient mice abrogate these effects, and these mice are more susceptible to BCG infection than wild-type mice.

A model summarizes the results of our study and indicates that the acid sphingomyelinase (Asm)/ceramide system is important in the control of BCG infection (Figure 8).

## 4. Discussion

Although it has been reported that both acid sphingomyelinase (Asm), and ROS are involved in mycobacterial infection [11,19,21], and that Asm-derived ceramide binds to and activates cathepsin D (CTSD) [16], our work, for the first time, suggests a novel mechanistic link between acid sphingomyelinase, cathepsin D, and ROS in mycobacterial infection (Figure 8) and indicates a pathogen-triggered signaling cascade that leads to bacterial clearance in vitro and in vivo.

A recent study indicated that Asm-mediated maturation of phagosomes is important for controlling mycobacterial infection [19]. Sortilin (*Sort1*), also named neurotensin receptor 3, is a transmembrane receptor that transports lysosomal proteins from the trans-Golgi network into lysosomes. Sortilin is upregulated during infection of macrophages with mycobacteria and is required for the delivery of both prosaposin and Asm from the Golgi complex to phagosomes. Studies on *Sort1*-deficient macrophages revealed a reduced association of Asm with phagosomes in *Sort1*-deficient cells compared to wild-type (wt) macrophages [20]. In vivo, *Sort1*-deficient mice exhibited substantially increased cellular infiltration of neutrophils and higher bacterial burden after infection with *Mycobacterium tuberculosis* in the lungs, suggesting that *Sort1*-dependent delivery of Asm into phagosomes is crucial for restricting bacterial growth [19].

Further studies demonstrated that Asm is required for the proper fusion of late phagosomes with lysosomes, which is crucial for efficient transfer of lysosomal antibacterial hydrolases into phagosomes [11]. Additional studies on zebrafish implied that Asm together with mitochondrial cyclophilin D induces necroptosis of macrophages upon infection with *Mycobacterium tuberculosis*, which might contribute to enhanced accessibility to the innate and specific immune system and thereby the elimination of intracellular mycobacteria [21].

Recognition of microbial pathogens by specific cell surface receptors and their internalization by professional macrophages is the first line of defense in bacterial infection [31]. Previous work has linked Asm with phagocytosis, reporting either involvement in bacterial internalization or maturation of phagosomes and lysosomes [32,34]. However, using a different range of assays, we show here that both binding and internalization of *Mycobacterium bovis* Bacillus Calmette-Guérin (BCG) into macrophages are independent of Asm. Neither the initial steps of phagosome maturation, such as Rab7 or Lamp1 expression, nor the localization of BCG in bone-marrow-derived macrophages differed between wt and Asm-deficient macrophages. Our flow cytometry studies employing Trypan blue quenching of adherent GFP-BCG confirm that the higher bacterial load we observed in Asm-deficient cells was not caused by defective BCG internalization by macrophages. This implies that Asm is not involved in adherence and internalization, at least in our model.

Following the uptake of microbes by phagocytes, bacteria containing phagosomes mature into phagolysosomes by gaining bactericidal factors such as active cathepsins to degrade microbes. A common defense mechanism used by mycobacteria is disruption of phagosome maturation and blockade of phagosomal acidification. It has been shown that *Mycobacterium tuberculosis* infection induces a general downregulation of cathepsins B, D, and S within macrophages, favoring increased intracellular survival of the pathogen [19,33]. Other studies suggested that Asm-derived ceramide specifically binds to cathepsin D (CTSD), resulting in enhanced enzymatic activity and proteolytic activation of proteins to be secreted [16].

Our presented results show that the expression of mature cathepsin D (CTSD) in wild-type (wt) macrophages was constitutively high and absent in cells with acid sphingomyelinase (Asm) deficiency, while other cathepsins, such as B, L, K, and S, did not differ between wt and Asm-deficient macrophages. The results also reveal that CTSD was relevant for the killing of BCG in macrophages as the downregulation/inhibition of mature cathepsin D increased bacterial load in wt macrophages to the level of Asm-deficient macrophages. In addition, immunofluorescence stainings for CTSD and bacteria in wt macrophages showed a higher co-localization between BCG and CTSD than in Asm-deficient cells. These results indicate that while CTSD expression/maturation was not modulated in vitro with BCG infection, it was regulated by acid sphingomyelinase (Asm) and was required to target BCG to CTSD and properly kill bacteria. Instead, in vivo the expression/maturation of CTSD in the liver of wt mice was significantly upregulated after BCG infection, whereas it was absent in the liver of Asm-deficient mice. These data support the in vitro results with macrophages and suggest that Asm controls CTSD, a process that leads to the killing of bacteria. The function of CTSD as a mediator of mycobacterial antigen presentation in macrophages may explain our finding that Asm-deficient mice were unable to control BCG infection. Due to a lack or delay of antigen presentation in Asm-deficient macrophages, the adaptive immune response in vivo may be retarded.

Granulomas, organized aggregates of immune cells, form in response to persistent stimuli/bacteria and are hallmarks of tuberculosis. Tuberculosis granulomas are most likely to be considered as host-protective structures formed to contain infection [35]. This notion is in accordance with our observation that in Asm-deficient mice, which were unable to clear BCG infection, hardly any granulomas were detectable. Since we observed no effect of Asm on the in vitro clustering of bone-marrow-derived macrophages after 24 h of infection (not shown), we assume that the in vivo granulomas could be affected by some other cell types, as mycobacterial granulomas are organized aggregates from different cell groups, including mature macrophages, differentiated or epithelial macrophages, foamy macrophages, and multinucleated (or Langerhans) giant cells [36].

Real-time observations of zebrafish embryos that were still at a stage prior to the appearance of T-lymphocytes revealed that *Mycobacterium marinum* induced granuloma formation could be initiated with macrophages alone [37]. In addition, studies with an in vitro model of mycobacterial granulomas, which enable cellular and molecular analysis of the very first steps in the host granulomatous response, indicated that macrophages were sufficient for the early stages of granuloma formation [38]. On the other hand, Ramakrishnan’s group found that primary granulomas promoted early dissemination of infection via egress of infected macrophages, thus promoting infection [39].

The increased susceptibility of Asm-deficient mice to BCG infection was reversed by transplantation with wt bone-marrow-derived macrophages in liver, suggesting that the reduced bacterial killing of Asm-deficient mice was due to an impaired microbicidal effect of Asm-deficient macrophages. In comparison to wt mice, the bacterial burden in the livers of Asm-deficient mice was enhanced after acute and chronic infection with BCG, whereas the bacterial load in spleens differed dramatically only after a short time of infection. Accordingly, the bacterial burden of Asm-deficient mice was significantly reduced after transplantation of wt macrophages in the liver and normalized to the level in wt mice, whereas the transplantation did not reverse susceptibility to BCG in the spleens of Asm-deficient mice. This discrepancy might be explained by different immune responses in different tissues.

A study that compared detailed phenotypical and functional analyses of murine Kupffer cells and splenic/peritoneal macrophages under steady-state conditions showed that liver macrophages exerted potent endocytic activity and displayed relatively high basal levels of ROS compared with splenic and peritoneal macrophages. Additional, ligation of TLR4, TLR7/8, and TLR9 on Kupffer cells resulted in a weak induction of IL-10; low or undetectable levels of IL-12, p40, and TNFα; and upregulation of CD40 on the surface compared to other macrophages [40]. These results suggest that Kupffer cells are specialized as phagocytes but only play a limited immune-regulatory role [35]. Bone-marrow-derived macrophages have been reported to have similar biological functions to Kupffer cells [41]. Compared to splenic macrophages, bone-marrow-derived macrophages show a stronger capacity for proliferation and phagocytosis [42]. Another aspect is that liver dendritic cells are generally weak activators of immunity, although they are capable of producing inflammatory cytokines, and certain subtypes potently activate T cells [42]. A previous study showed that liver dendritic cells were less mature, captured less antigen, and induced less T cell stimulation than spleen dendritic cells because of differences of subtype composition [43]. A study based on an investigation of in situ expression of MHC class II on liver dendritic cells identified that murine liver dendritic cells did not display measurable levels of the T cell-costimulatory molecules CD40, CD80, and CD86, which implied a low immune-stimulatory capacity of liver dendritic cells [44]. Kupffer cells have been shown to play a critical role in mycobacterial infections. A recent study suggested that mouse Kupffer cells, in comparison to alveolar macrophages, are able to better restrict the growth of *Mycobacterium tuberculosis*, because they are more capable of producing cytokines and molecules that modulate autophagy and cytoskeleton than dendritic cells [45]. Together, these studies suggest different mechanisms of regulating immune responses in liver and spleen than our model. This also implies that other tissue immune cells, such as dendritic cells or T-lymphocytes, may have important functions predominantly in the spleen, while the immune response to BCG in the liver is mostly driven by macrophages.

## 5. Conclusions

The current study suggests rapid control of early *Mycobacterium bovis* Bacillus Calmette-Guérin (BCG) infection in wild-type mice and less efficient bacterial killing upon infection in mice that are deficient for acid sphingomyelinase. Infection of wild-type macrophages resulted in activation of acid sphingomyelinase, which increased reactive oxygen species (ROS) via nicotinamide adenine dinucleotide phosphate (NADPH) oxidase subunit p47phox. ROS promote BCG degradation by cathepsin D. Deficiency of acid sphingomyelinase in macrophages prevents these effects. Transplantation of wild-type macrophages into acid-sphingomyelinase-deficient mice completely reversed the susceptibility of acid-sphingomyelinase-deficient mice to BCG, elucidating the role of macrophages in the immune defense, particularly of the liver. These findings indicate that the acid sphingomyelinase–ceramide system is important in the control of BCG infection.

## Supplementary Materials

Supplementary data for this paper can be found online at: https://www.mdpi.com/2073-4409/9/11/2406/s1.

## Abbreviations

BCG, *Mycobacterium bovis* Bacillus Calmette-Guérin; Asm, acid sphingomyelinase; CTSD, cathepsin D; ROS, reactive oxygen species; BMDMs, bone-marrow-derived macrophages; CFU, colony-forming units; NADPH, nicotinamide adenine dinucleotide phosphate; wt, wild-type.

## References

[1] Hannun Y.A., Obeid L.M. (2008). Principles of bioactive lipid signalling: Lessons from sphingolipids. Nat. Rev. Mol. Cell. Biol..

[2] Kolesnick R.N., Haimowitz-Friedman A., Fuks Z. (1994). The sphingomyelin signal transduction pathway mediates apoptosis for tumor necrosis factor, Fas, and ionizing radiation. Biochem. Cell. Biol..

[3] Schütze S., Potthoff K., Machleidt T., Berkovic D., Wiegmann K., Krönke M. (1992). TNF activates NF-kappa B by phosphatidylcholine-specific phospholipase C-induced “acidic” sphingomyelin breakdown. Cell.

[4] Grassmé H., Jekle A., Riehle A., Schwarz H., Berger J., Sandhoff K., Kolesnick R., Gulbins E. (2001). CD95 signaling via ceramide-rich membrane rafts. J. Biol. Chem..

[5] Grassmé H., Gulbins E., Brenner B., Ferlinz K., Sandhoff K., Harzer K., Lang F., Meyer T.F. (1997). Acidic sphingomyelinase mediates entry of *N. gonorrhoeae* into nonphagocytic cells. Cell.

[6] Esen M., Schreiner B., Jendrossek V., Lang F., Fassbender K., Grassmé H., Gulbins E. (2001). Mechanisms of *Staphylococcus aureus* induced apoptosis of human endothelial cells. Apoptosis.

[7] Grassmé H., Jendrossek V., Riehle A., von Kürthy G., Berger J., Schwarz H., Weller M., Kolesnick R., Gulbins E. (2003). Host defense against *Pseudomonas aeruginosa* requires ceramide-rich membrane rafts. Nat. Med..

[8] Utermöhlen O., Karow U., Lohler J., Krönke M. (2003). Severe impairment in early host defense against *Listeria monocytogenes* in mice deficient in acid sphingomyelinase. J. Immunol..

[9] McCollister B.D., Myers J.T., Jones-Carson J., Völker D.R., Vázquez-Torres A. (2007). Constitutive acid sphingomyelinase enhances early and late macrophage killing of *Salmonella enterica* serovar Typhimurium. Infect. Immun..

[10] Falcone S., Perrotta C., De Palma C., Pisconti A., Sciorati C., Capobianco A., Rovere-Querini P., Manfredi A.A., Clementi E. (2004). Activation of acid sphingomyelinase and its inhibition by the nitric oxide/cyclic guanosine 3′,5′-monophosphate pathway: Key events in *Escherichia coli*-elicited apoptosis of dendritic cells. J. Immunol..

[11] Utermöhlen O., Herz J., Schramm M., Krönke M. (2008). Fusogenicity of membranes: The impact of acid sphingomyelinase on innate immune responses. Immunobiol..

[12] Avota E., Gulbins E., Schneider-Schaulies S. (2011). DC-SIGN mediated sphingomyelinase-activation and ceramide generation is essential for enhancement of viral uptake in dendritic cells. PLoS Pathog..

[13] Miller M.E., Adhikary S., Kolokoltsov A.A., Davey R.A. (2012). Ebolavirus requires acid sphingomyelinase activity and plasma membrane sphingomyelin for infection. J. Virol..

[14] Majumder S., Dey R., Bhattacharjee S., Rub A., Gupta G., Bhattacharyya Majumdar Saha B., Majumdar S. (2012). Leishmania-induced biphasic ceramide generation in macrophages is crucial for uptake and survival of the parasite. J. Infect. Dis..

[15] Zhang Y., Li X., Carpinteiro A., Gulbins E. (2008). Acid sphingomyelinase amplifies redox signaling in *Pseudomonas aeruginosa*-induced macrophage apoptosis. J. Immun..

[16] Heinrich M., Wickel M., Schneider-Brachert W., Sandberg C., Gahr J., Schwander R., Weber T., Saftig P., Peters C., Brunner J. (1999). Cathepsin D targeted by acid sphingomyelinase-derived ceramide. EMBO J..

[17] WHO Global Tuberculosis Report 2016. WHO Report 2016. http://www.whoint/tb/publications/global_report/en/.

[18] Anes E., Kühnel M.P., Bos E., Moniz-Péreira J., Habermann A., Griffiths G. (2003). Selected lipids activate phagosome actin assembly and maturation resulting in killing of pathogenic mycobacteria. Nat. Cell. Biol..

[19] Vázquez C.L., Rodgers A., Herbst S., Coade S., Gronow A., Guzman C.A., Wilson M.S., Kanzaki M., Nykjaer A., Gutierrez M.G. (2016). The proneurotrophin receptor sortilin is required for *Mycobacterium tuberculosis* control by macrophages. Sci. Rep..

[20] Wahe A., Kasmapour B., Schmaderer C., Liebl D., Sandhoff K., Nykjaer A., Griffiths G., Gutiérrez M.G. (2010). Golgi-to-phagosome transport of acid sphingomyelinase and prosaposin is mediated by sortilin. J. Cell. Sci..

[21] Roca F.J., Ramakrishnan L. (2013). TNF dually mediates resistance and susceptibility to mycobacteria via mitochondrial reactive oxygen species. Cell.

[22] Horinouchi K., Erlich S., Perl D.P., Ferlinz K., Bisgaier C.L., Sandhoff K., Desnick R.J., Stewart C.L., Schuchman E.H. (1995). Acid sphingomyelinase deficient mice: A model of types A and B Niemann-Pick disease. Nat Genet..

[23] Carpinteiro A., Becker K.A., Japtok L., Hessler G., Keitsch S., Pozgajova M., Schmid K.W., Adams C., Müller S., Kleuser B. (2015). Regulation of hematogenous tumor metastasis by acid sphingomyelinase. EMBO Mol. Med..

[24] Fazal N., Lammas D.A., Raykundalia C., Bartlett R., Kumararatne D.S. (1992). Effect of blocking TNF-alpha on intracellular BCG (Bacillus Calmette Guerin) growth in human monocyte-derived macrophages. FEMS Microbiol. Immunol..

[25] Humphreys I.R., Stewart G.R., Turner D.J., Patel J., Karamanou D., Snelgrove R.J., Young D.B. (2006). A role for dendritic cells in the dissemination of mycobacterial infection. Microbes Infect..

[26] Hed J. (1986). Methods for distinguishing ingested from adhering particles. Meth. Enzymol..

[27] Szollosi J., Tron L., Damjanovich S., Helliwell S.H., Arndt-Jovin D., Jovin T.M. (1984). Fluorescence energy transfer measurements on cell surfaces: A critical comparison of steady-state fluorimetric and flow cytometric methods. Cytometry.

[28] Mühle C., Kornhuber J. (2017). Assay to measure sphingomyelinase and ceramidase activities efficiently and safely. J. Chromatogr..

[29] Abais J.M., Xia M., Li G., Gehr T.W., Boini K.M., Li P.L. (2014). Contribution of endogenously produced reactive oxygen species to the activation of podocyte NLRP3 inflammasomes in hyperhomocysteinemia. Free Radic. Biol. Med..

[30] Xu M., Xia M., Li X.X., Han W.Q., Boini K.M., Zhang F., Zhang Y., Ritter J.K., Li P.-L. (2012). Requirement of translocated lysosomal V1 H(+)-ATPase for activation of membrane acid sphingomyelinase and raft clustering in coronary endothelial cells. Mol. Biol. Cell.

[31] Weiss G., Schaible U.E. (2015). Macrophage defense mechanisms against intracellular bacteria. Immunol. Rev..

[32] Li C., Wu Y., Riehle A., Orian-Rousseau V., Zhang Y., Gulbins E., Grassmé H. (2017). Regulation of *Staphylococcus aureus* infection of macrophages by CD44, reactive oxygen species, and acid sphingomyelinase. Antioxid. Redox. Sign..

[33] Pires D., Marques J., Pombo J.P., Carmo N., Bettencourt P., Nevrolles O., Lugo-Villarino G., Anes E. (2016). Role of cathepsins in *Mycobacterium tuberculosis* survival in human macrophages. Sci. Rep..

[34] Schramm M., Herz J., Haas A., Krönke M., Utermöhlen O. (2008). Acid sphingomyelinase is required for efficient phago-lysosomal fusion. Cell. Microbiol..

[35] Ulrichs T., Kaufmann S.H.E. (2006). New insights into the function of granulomas in human tuberculosis. J. Pathol..

[36] Adams D.O. (1976). The granulomatous inflammatory response. Am. J. Pathol..

[37] Davis J.M., Clay H., Lewis J.L., Ghori N., Herbomel P., Ramakrishnan L. (2002). Real-time visualization of mycobacterium-macrophage interactions leading to initiation of granuloma formation in zebrafish embryos. Immunity.

[38] Puissegur M.P., Lay G., Gilleron M., Botella L., Nigou J., Marrakchi H., Mari B., Duteyrat J.L., Guerardel Y., Kremer L. (2007). Mycobacterial lipomannan induces granuloma macrophage fusion via a TLR2-dependent, ADAM9- and beta1 integrin-mediated pathway. J. Immunol..

[39] Davis J.M., Ramakrishnan L. (2009). The role of the granuloma in expansion and dissemination of early tuberculous infection. Cell.

[40] Movita D., Kreefft K., Biesta P., van Oudenaren A., Leenen P.J., Janssen H.L., Boonstra A. (2012). Kupffer cells express a unique combination of phenotypic and functional characteristics compared with splenic and peritoneal macrophages. J. Leuk. Biol..

[41] Beattie L., Sawtell A., Mann J., Frame T.C.M., Teal B., de Labastida Rivera F., Brown N., Walwyn-Brown K., Moore J.W.J., MacDonald S. (2016). Bone marrow-derived and resident liver macrophages display unique transcriptomic signatures but similar biological functions. J. Hepatol..

[42] Wang C., Yu X., Cao Q., Wang Y., Zheng G., Tan T.K., Zhao H., Zhao Y., Wang Y., Harris D. (2013). Characterization of murine macrophages from bone marrow, spleen and peritoneum. BMC Immunol..

[43] Pillarisetty V.G., Shah A.B., Miller G., Bleier J.I., DeMatteo R.P. (2004). Liver dendritic cells are less immunogenic than spleen dendritic cells because of differences in subtype composition. J. Immunol..

[44] Inaba K., Witmer-Pack M., Inaba M., Hathcock K.S., Sakuta H., Azuma M., Yagita H., Okumura K., Linsley P.S., Ikehara S. (1994). The tissue distribution of the B7-2 costimulator in mice: Abundant expression on dendritic cells in situ and during maturation in vitro. J. Exp. Med..

[45] Thandi R.S., Tripathi D., Radhakrishnan R.K., Paidipally P., Vankayalapati R. (2018). Kupffer cells restricts *Mycobacterium tuberculosis* growth better than alveolar macrophages. J. Immunol..

