# Supplementary material for: Acid Sphingomyelinase Contributes to the Control of Mycobacterial Infection via a Signaling Cascade Leading from Reactive Oxygen Species to Cathepsin D
Source: Cells. 2020 Nov 3;9(11):2406. doi: 10.3390/cells9112406 (PMC7693114; doi:10.3390/cells9112406)
Supplement: Supplementary file 1 [file cells-09-02406-s001.pdf]

Supplementary material to:

Acid Sphingomyelinase Contributes to the Control of Mycobacterial Infection via a Signaling Cascade Leading from Reactive Oxygen Species to Cathepsin D

Yuqing Wu, Cao Li, Huiming Peng, Ashraf Swaidan, Andrea Riehle, Barbara Pollmeier , Yang Zhang, Erich Gulbins, Heike Grassmé

3.1. Acid sphingomyelinase-deficient mice are more susceptible to BCG-infection than wild-type mice

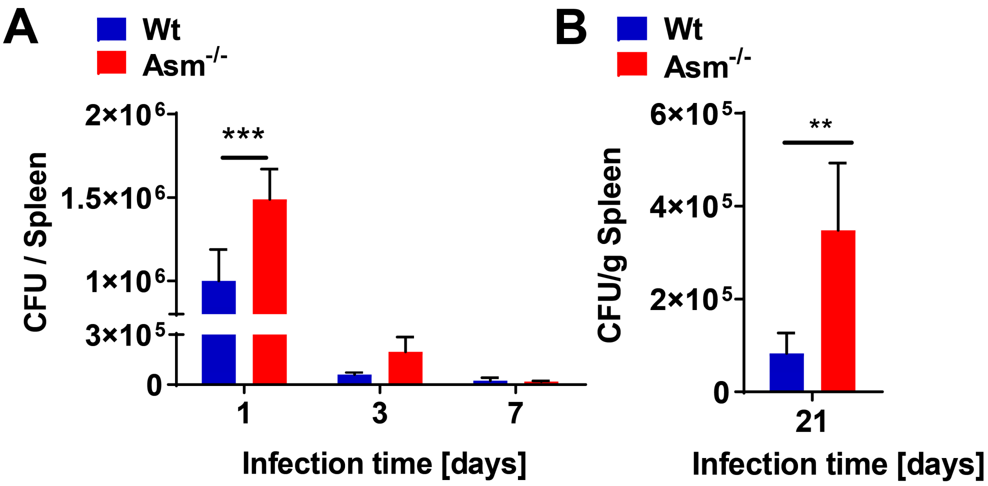

Figure S1: Acid sphingomyelinase-deficient mice are more susceptible to BCG-infection than wild-type mice

(A,B) The total number of BCG in spleen tissue homogenates was determined by CFU assays at 1, 3, 7 or 21 days (d) after infection. Shown are means ± SD of bacterial numbers of 6 independent experiments, p-values are given by ANOVA followed by Bonferroni's multiple comparisons test. Wt = Wild-type, Asm-/- = acid sphingomyelinase deficient.

3.2. Acid sphingomyelinase-deficiency impairs BCG killing but not internalization by macrophages

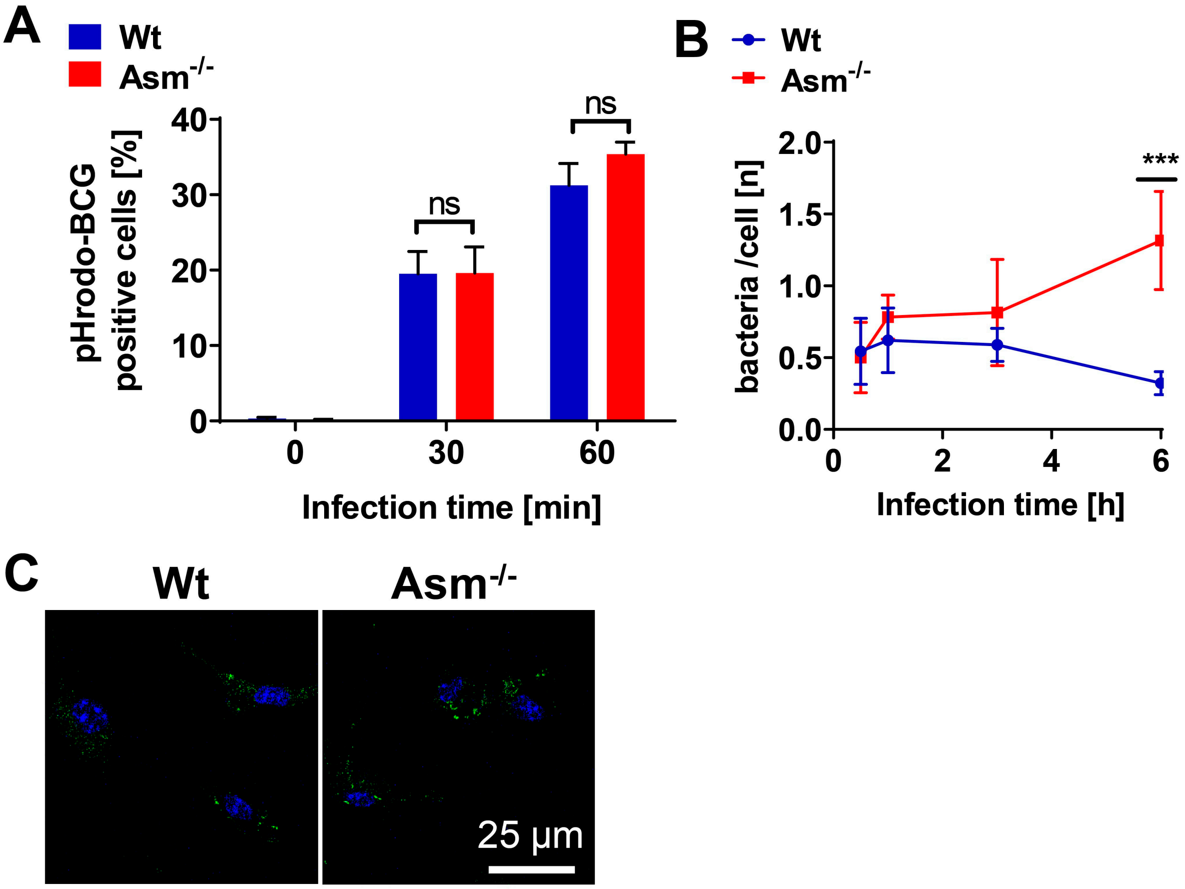

Figure S2: Acid sphingomyelinase-deficiency impairs BCG killing but not internalization by macrophages

(A) Bone marrow-derived macrophages were infected for the indicated time with pHrodo-red pre-stained BCG, which show red fluorescence after internalization and analyzed by FACS at BL2 channel. Uninfected cells were used as a control. Results are representative of 3 independent studies. **(B)** Macrophages were infected with bacteria for the indicated time. The number of GFP-BCG in infected cells were quantified by microscope by counting GFP expressing BCG with a 100x lens. 50 cells were counted in each experiment and the results are representative of 3 independent experiments. Results are the mean ± SD. Wt = Wild-type, Asm-/- = acid sphingomyelinase deficient. **(C)** Wt or Asm-deficient macrophages were infected with GFP-BCG (green) for 6 h, fixed and were mounted on glass coverslips with ROTI®Mount FluorCare DAPI (Roth). Samples were analyzed by confocal microscopy with a 100x lens lens. Shown is a picture representing 3 independent studies. Scale bar = 25 µm.

3.2. Acid sphingomyelinase-deficiency impairs BCG killing but not internalization by macrophages

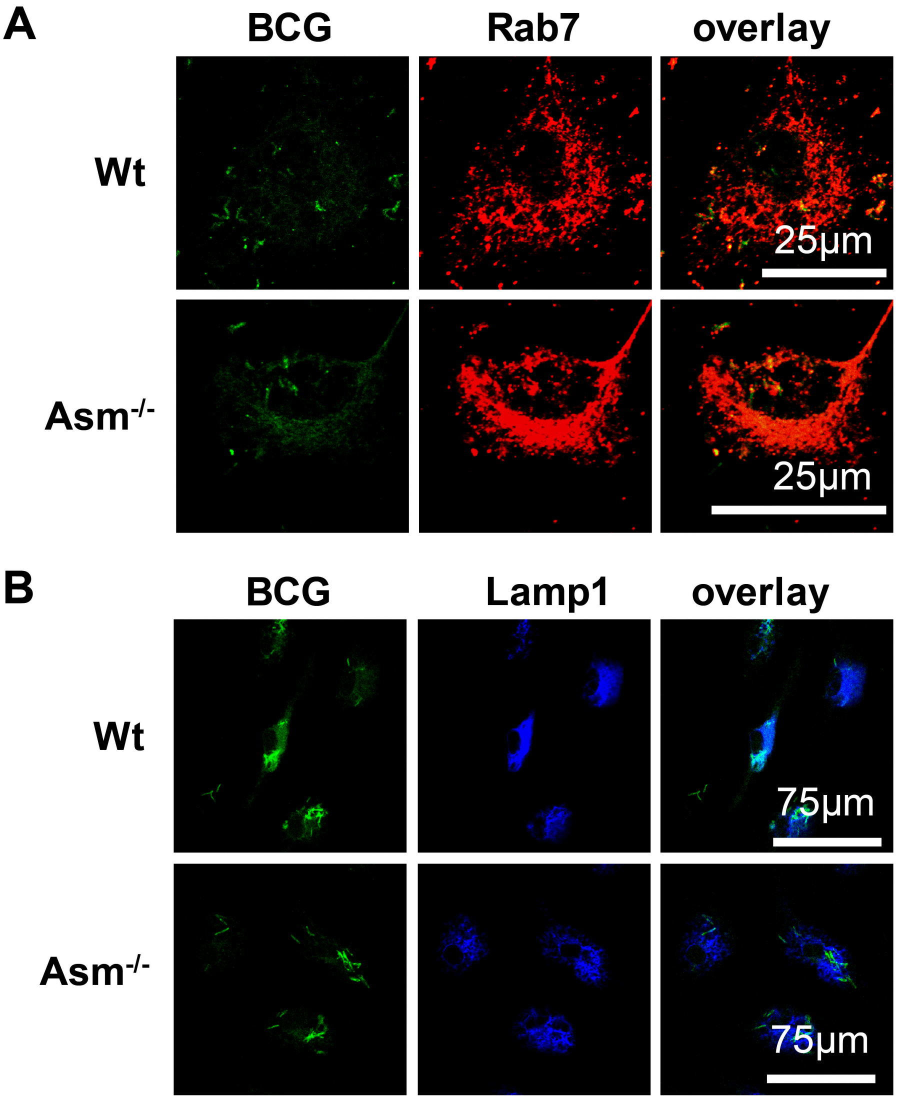

Figure S3: Acid sphingomyelinase-deficiency impairs BCG killing but not internalization by macrophages

Bone marrow-derived macrophages were left uninfected or infected with BCG for 60 min, fixed and stained with **(A)** Cy3-coupled Rab7 and **(B)** Cy5-coupled Lamp1. Samples were analyzed by confocal microscopy. Shown are pictures representing 3 independent studies. Scale bar = 25 µm.

3.3. Acid sphingomyelinase determines expression of cathepsin D, which is essential for BCG degradation

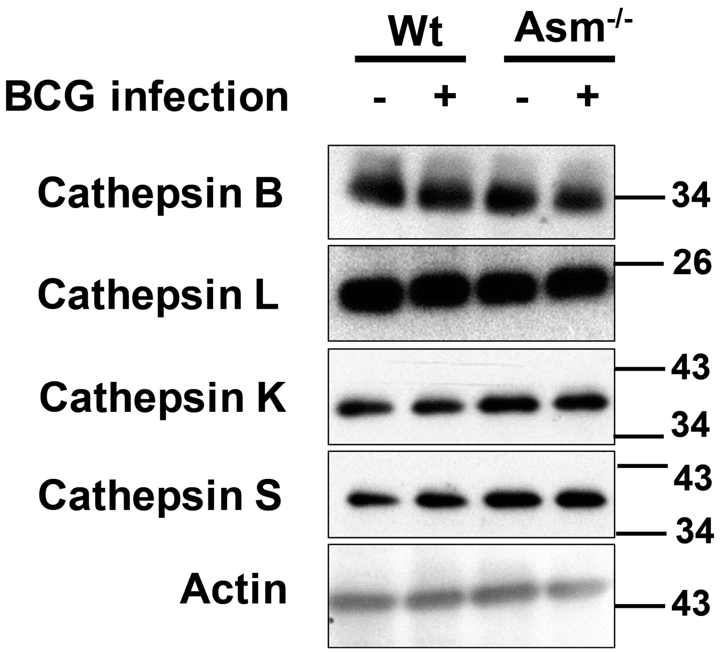

Figure S4: **Acid sphingomyelinase determines expression of cathepsin D, which is essential for BCG degradation**

Bone marrow-derived macrophages were infected with BCG for the indicated periods of time and were then subjected to Western blot analysis using antibodies of cathepsin B, L, K, S and β-actin (antibodies against cathepsin B were from R&D, against cathepsin L/K/S from santa cruz). Shown are blots representing 3 independent studies.

3.4. Acid sphingomyelinase controls ROS production upon BCG infection via NADPH oxidase

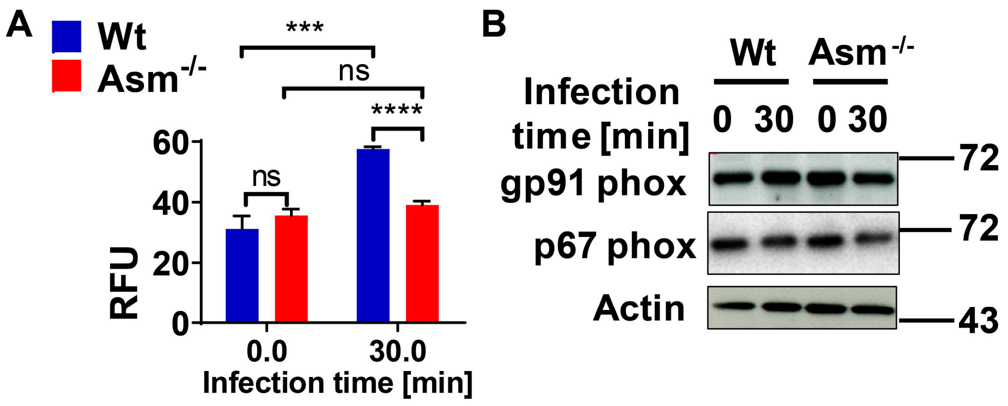

Figure S5. Acid sphingomyelinase controls ROS production upon BCG infection via nicotinamide adenine dinucleotide phosphate (NADPH) oxidase.

Wild-type (Wt) or acid sphingomyelinase deficient (Asm-/-) bone marrow-derived macrophages (BMDMs) were infected with BCG for the indicated time. **(A)** Cells were incubated with a fluorescence probe, ROS deep red dye. The fluorescence was determined by a fluorescence microplate reader at Ex/Em=650/675 nm (cut off 665 nm) after 30 min. A relative fluorescence unit (RFU) was used to represent the ROS release. Displayed is the mean ± SD of 3 experiments, ANOVA and Bonferroni's multiple comparisons test. **(B)** Expression of the NADPH oxidase subunit p67 phox and gp91 phox were determined by western blotting of lysates obtained from BCG-infected or non-infected macrophages.

3.7. Transplantation of wild-type macrophages reverses susceptibility of acid sphingomyelinase-deficient mice to BCG infection

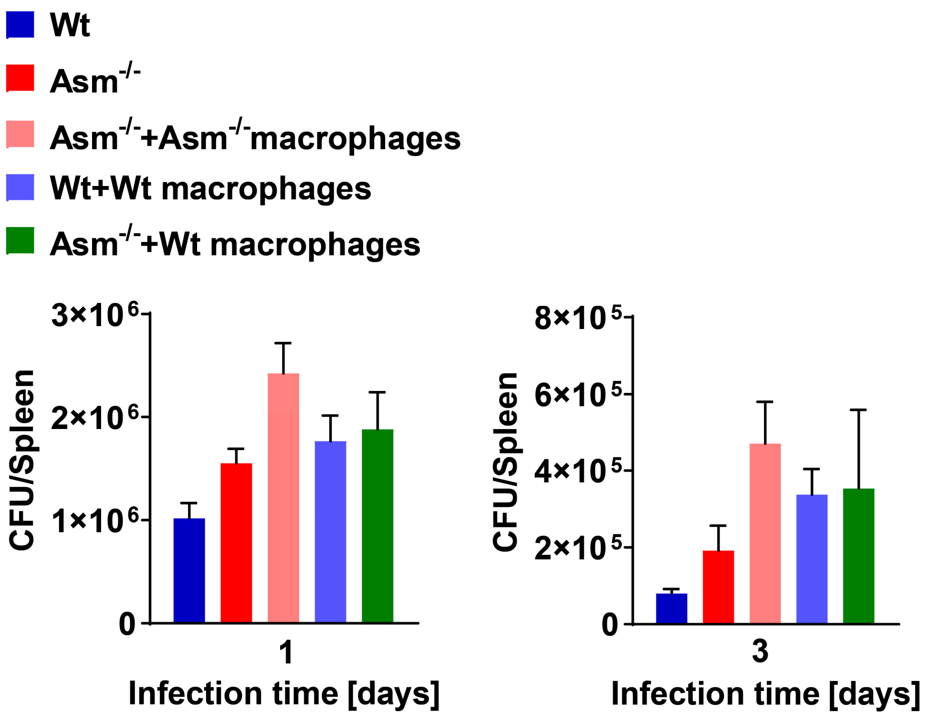

Figure S6: Transplantation of wildtype macrophages reverses susceptibility of acid sphingomyelinase-deficient mice to BCG infection

Wild-type (Wt) or acid sphingomyelinase-deficient (Asm-/-) mice were intravenously injected with clodronate liposomes 2 days before infection and were transplanted with 5×10^6 Wt or Asm-/- bone marrow-derived macrophages via intravenous injection 1 day before infection or left untreated. Transplanted and control mice were infected with 10^7 BCG for 1 day or 3 days. The total numbers of BCG in in spleen homogenates at 1 and 3 days post infection by colony forming unit (CFU) assays. Shown are the mean ± SD. P-values were determined by one-way ANOVA followed by Bonferroni's multiple comparisons test, n=6.

Materials and methods

1. Method for pHrodo-BCG phagocytosis

For phagocytosis assays with pHrodo-BCG in bone-marrow-derived macrophages, BCG were pre-labeled with pHrodo phagocytosis particle labeling kit (Invitrogen) according to the manufactory instructions. Briefly, pHrodo Red succinimidyl ester was resuspended in DMSO to obtain a solution of about 10mM and stored at -20°C until use. Before infection, 10^8 BCG were resuspended in 200μL 1xPBS and incubated with 1μL pHrodo Red for 30 min at 30°C and washed once with 1xPBS. PHrodo positive cells were determined by Attune NxT flow cytometry and FlowJo software v10.

2. Staining of endosomal markers

Immunofluorescence stainings were performed, as described in the original paper. Lamp1-Alexa Fluor 647 conjugated antibodies were from BioLegend, Rab7 antibodies from Santa Cruz, second antibodies from Jackson Immuno Research.

3. Measurement of ROS production

ROS were measured by using the Cellular Reactive Oxygen Species Detection Assay Kit (Deep Red Fluorescence, Abcam) according to the manufacturer's instructions. Briefly, 10^4 cells were seeded in 96-well plate and infected with BCG for the indicated time. Cells were then washed with PBS, incubated with ROS deep red dye for 30 min and analyzed with a microplate reader at Ex/Em = 650/675 nm (cut off 665 nm) (SpectraMax Gemini EM, Molecular Devices GmbH, Germany).
